# Interplay between the HTLV-2 Tax and APH-2 proteins in the regulation of the AP-1 pathway

**DOI:** 10.1186/1742-4690-9-98

**Published:** 2012-12-03

**Authors:** Céline Marban, Áine McCabe, Terence N Bukong, William W Hall, Noreen Sheehy

**Affiliations:** 1 Centre for Research in Infectious Diseases, School of Medicine and Medical Science, University College Dublin, Belfield, Dublin 4, Ireland; 2 Inserm U977, Faculté de Chirurgie Dentaire, 1 Place de l'Hôpital, 67000, Strasbourg, France

**Keywords:** HTLV-2, APH-2, Tax2, AP-1, Jun

## Abstract

**Background:**

In contrast with human T-cell leukemia virus type 1 (HTLV-1) that causes ATL (adult T-cell leukemia), HTLV-2 has not been causally linked to malignant disease. The minus strand of the HTLV genomes encode the regulatory proteins HTLV-1 bZIP factor (HBZ) for HTLV-1 and antisense protein of HTLV-2 (APH-2) for HTLV-2. Unlike the viral proteins Tax1 and Tax2, both HBZ and APH-2 are constitutively expressed in infected cells suggesting that they may play important roles in the pathogenesis of these viruses. To date, very little is known about the function of APH-2 except that it inhibits Tax2-mediated transcription of HTLV-2 genes. In the present study, we investigated the role of APH-2 in basal and Tax2B-mediated activation of the AP-1 pathway.

**Results:**

We demonstrate that, unlike HBZ, APH-2 stimulates basal AP-1 transcription by interacting with c-Jun and JunB through its non-conventional bZIP domain. In addition, when Tax2 and APH-2 are co-expressed, they physically interact *in vivo* and *in vitro* and APH-2 acts as an inhibitor of Tax2-mediated activation of AP-1 transcription.

**Conclusions:**

This report is the first to document that HTLV-2 can modulate the AP-1 pathway. Altogether our results reveal that, in contrast with HBZ, APH-2 regulates AP-1 activity in a Tax2-dependant manner. As the AP-1 pathway is involved in numerous cellular functions susceptible to affect the life cycle of the virus, these distinct biological properties between HBZ and APH-2 may contribute to the differential pathogenic potential of HTLV-1 and HTLV-2.

## Background

Thirty years after the discovery of the first human oncogenic virus, the human T-cell leukemia virus (HTLV) family of retroviruses is now composed of four members: the well documented HTLV-1 and HTLV-2 and the recently discovered HTLV-3 and HTLV-4 [1-4]. HTLV-1 is the etiological agent of multiple disorders including adult T-cell leukemia (ATL) and HTLV-1-associated myelopathy/tropical spastic paraparesis (HAM/TSP) [5,6]. The role of HTLV-2 in human disease is less clearly defined but infection is associated with lymphocyte proliferation and high platelet counts as well as milder neurological disorders [7-9]. However, while not being associated with ATL like disorders, HTLV-2 is still able to efficiently induce transformation of primary T-cells [10].

In addition to the structural and enzymatic proteins common to all retroviruses, HTLV-1 also encodes regulatory proteins such as Tax1. The HTLV-1 Tax protein is a transcriptional activator that regulates HTLV-1 gene expression but also modulates the expression of numerous cellular genes through activation of cellular transcription factors including NF-κB [11], CREB [12-16], SRF [17] and AP-1 [18]. Activation of these major cellular signal transduction pathways plays a critical role in T-cell transformation, and therefore ATL development. Previous reports indicate that AP-1 activity is induced in ATL cells [18,19]. Moreover, HTLV-1 Tax up-regulates the transcription of several AP-1 family members such as c-Jun, JunD, c-Fos and Fra-1 [20,21].

AP-1 consists of a myriad of homo- or hetero- dimers that belong to the Jun, Fos, Maf and ATF subfamilies. All AP-1 family members harbour a basic leucine zipper (bZIP) motif, which consists of a DNA binding domain rich in basic amino acids adjacent to a leucine zipper structure required for protein-protein dimerization [22]. AP-1 dimers recognize either TPA response elements (TRE) or cAMP response elements (CRE) which are present in the promoter region of many cellular genes involved in a large spectrum of biological processes including cell proliferation, apoptosis and oncogenic transformation [23].

Transcription from the 3’ Long Terminal Repeat (LTR) of the HTLV genomes governs the expression of antisense regulatory proteins named HTLV-1 bZIP factor (HBZ) for HTLV-1 [24], antisense protein of HTLV-2 (APH-2) for HTLV-2 [25], APH-3 and APH-4 for HTLV-3 and HTLV-4, respectively [26]. The HBZ gene has been described as a key player in HTLV-1 pathogenesis as its expression appears to be critical for ATL development and disease severity in HAM/TSP [27-29]. HBZ contains a bZIP motif, which enables it to heterodimerize with cellular transcription factors in order to regulate viral or cellular transcription. Thus, by interacting with CREB, HBZ prevents the binding of CREB to the CRE in the HTLV-1 LTR, resulting in the inhibition of HTLV-1 gene transcription [30]. HBZ also interacts with the transcription factor ATF3, thus preventing its ability to enhance p53 transcriptional activity, and therefore the proliferation of ATL cells [31]. In addition, HBZ is able to inhibit the classical NF-κB pathway by binding p65 and therefore decreasing p65 DNA binding capacity, a mechanism used by the virus to escape from the host immune system [32]. Moreover, numerous studies have also reported that HBZ interacts with AP-1 members of the Jun subfamily such as c-Jun, JunB and JunD and modulates their transcriptional activity [33,34]. The interaction between HBZ and c-Jun as well as HBZ and JunB results in repression of c-Jun and JunB activity through degradation or sequestration into transcriptionally inactive nuclear bodies [35-38]. However, by interacting with JunD, HBZ can activate JunD-dependant transcription of cellular genes including the human telomerase reverse transcriptase [34,39].

The role of APH-2 in the pathogenesis of HTLV-2 infection is less defined. To date, only one study reveals that APH-2 does not promote lymphocytosis [40]. APH-2 harbours a non-conventional bZIP motif as it displays seven instead of six amino acids between the sixth and the seventh leucine. Despite the lack of a classic bZIP domain, APH-2 is still able to interact with CREB and repress Tax2-mediated transcription activation of HTLV-2 genes [25].

In the present report, we investigated the role of HTLV-2 proteins APH-2 and Tax2B on AP-1 activity. We demonstrated that APH-2 interacts with c-Jun and JunB through its non-canonical bZIP domain and enhances their ability to activate AP-1 transcription. Surprisingly, when APH-2 and Tax2B are co-expressed, APH-2 binds Tax2B and acts as a repressor of Tax2B-mediated activation of AP-1 transcription.

Taken together, our results reveal that both APH-2 and Tax2 act as transcription factors that subtly regulate AP-1 transcription. These findings strongly suggest that APH-2 and Tax2 are involved in the regulation of many biological processes involving AP-1 and therefore indirectly help the virus to replicate and/or counteract the host’s immune system.

## Results

### APH-2 stimulates the transcriptional activity of c-Jun, JunB and JunD

**Figure 1:**
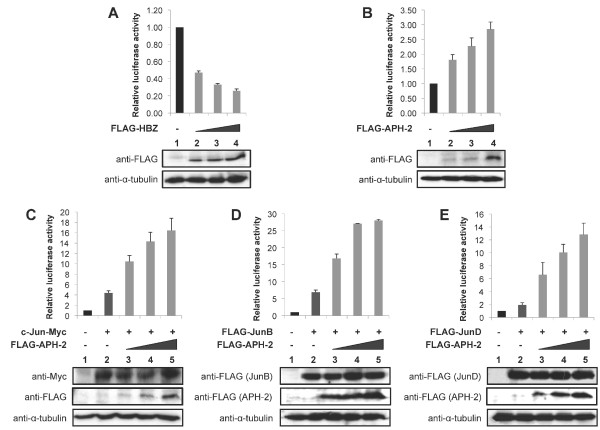
**APH-2 stimulates basal AP-1 transcription through c-Jun, JunB and JunD.** 293T cells were transfected with the pAP-1 luciferase construct and the indicated expression vectors. Luciferase and Renilla activities were measured 48 hours after transfection. The average of at least three different experiments is shown. The expression levels of the transfected proteins were analysed by Western blot using the indicated antibodies. (**A**) HBZ inhibits AP-1 transcription. (**B**) APH-2 activates AP-1-mediated transcription. (**C**, **D** and **E**) APH-2 enhances the stimulatory effect of c-Jun, JunB and JunD, respectively, on an AP-1 responsive promoter.

In order to investigate the effect of APH-2 on basal AP-1 transcription compared to HBZ, we performed luciferase assays using an AP-1 *cis*-reporter plasmid that contains the luciferase reporter gene driven by a basic promoter element plus seven repeats of AP-1 binding sites. We transfected the luciferase reporter construct together with increasing amounts of HBZ or APH-2 expression vectors into 293T cells (Figure 1A and 1B, upper panels). The expression levels of the transfected proteins were confirmed by Western blot (Figure 1A and 1B, lower panels). Interestingly, while HBZ inhibits AP-1-mediated transcription (Figure 1A, columns 2–4), APH-2 stimulates basal AP-1 transcription in a dose dependant manner (Figure 1B, columns 2–4).

Previous studies have shown that HBZ affects AP-1 transcription by modulating the transcriptional activity of all members of the Jun subfamily. On the one hand, HBZ represses transcription mediated by c-Jun and JunB while it activates JunD-dependant transcription [34,35,37,38]. To examine whether APH-2-mediated activation of AP-1 transcription results in the stimulation of Jun activity, we performed luciferase assays. Cells were co-transfected with the AP-1-Luc reporter construct, c-Jun, JunB or JunD as well as APH-2 expression vectors (Figure 1C, 1D and 1E, respectively, upper panels). Western blot analysis demonstrated that FLAG-APH-2 does not affect the levels of overexpressed c-Jun-Myc, FLAG-JunB and FLAG-JunD (Figure 1C, 1D and 1E, respectively, lower panels). As expected, in the absence of APH-2 expression, c-Jun (Figure 1C, column 2), JunB (Figure 1D, column 2) and JunD (Figure 1E, column 2) activate AP-1 transcription. Interestingly, co-expression of APH-2 further enhances c-Jun (Figure 1C, columns 3–5), JunB (Figure 1D, columns 3–5) and JunD-mediated (Figure 1E, columns 3–5) transactivation.

These results collectively reveal that APH-2 is a co-activator of c-Jun, JunB and JunD.

### APH-2 interacts with c-Jun and JunB but not JunD

**Figure 2:**
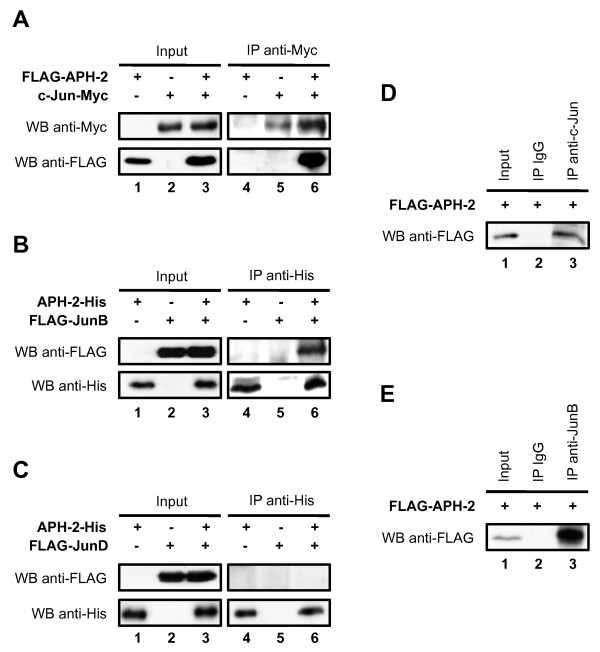
**APH-2 interacts with c-Jun and JunB *in vivo.*** 293T cells were transiently transfected with the indicated expression plasmids. Two days after transfection, nuclear extracts were immunoprecipitated with the indicated antibodies (IP). The presence of proteins of interest in the immunoprecipitates was visualized by Western blot using the indicated antibodies (WB). (**A**) APH-2 interacts with c-Jun. (**B**) APH-2 binds JunB. (**C**) APH-2 does not interact with JunD. (**D**) APH-2 associates with endogenous c-Jun. (**E**) APH-2 associates with endogenous JunB.

To further decipher the molecular mechanisms in the property of APH-2 to activate Jun-mediated transcription, we tested whether APH-2 interacts with c-Jun, JunB and JunD *in vivo*. First, we co-transfected FLAG-APH-2 and/or c-Jun-Myc expression vectors in 293T cells as indicated, and the nuclear extracts were subjected to co-immunoprecipitation (Figure 2A). FLAG antibodies were able to detect FLAG-APH-2 in the nuclear extracts from cells overexpressing FLAG-APH-2 and c-Jun-Myc and immunoprecipitated with Myc antibodies (Figure 2A, WB anti-FLAG, column 6). However, no signal was obtained when FLAG-APH-2 or c-Jun-Myc were overexpressed alone (Figure 2A, WB anti-FLAG, columns 4 and 5). These results suggest that APH-2 interacts with c-Jun *in vivo*. To test whether APH-2 interacts with JunB and JunD, nuclear extracts from 293T cells overexpressing APH-2-His and/or FLAG-JunB (Figure 2B) or APH-2-His and/or FLAG-JunD (Figure 2C) were immunoprecipitated with His antibodies. FLAG antibodies were able to detect FLAG-JunB only in nuclear extracts overexpressing both APH-2-His and FLAG-JunB confirming that APH-2 interacts with JunB (Figure 2B, WB anti-FLAG, columns 4–6). Surprisingly, FLAG-JunD was not detected either in the immunoprecipitated nuclear extracts overexpressing APH-2-His alone, FLAG-JunD alone or both APH-2-His and FLAG-JunD indicating that APH-2 is unable to bind JunD (Figure 2C, WB anti-FLAG, columns 4–6). Reciprocal co-immunoprecipitations confirmed the interaction of APH-2 with c-Jun and JunB but not JunD (Additional file 1A, 1B and 1C, respectively).

To further characterize the interaction between APH-2 and c-Jun/JunB, we tested whether APH-2 also associates with endogenous c-Jun and JunB. We, therefore, co-immunoprecipitated endogenous c-Jun and JunB from nuclear extracts of FLAG-APH-2 transfected cells. As shown in Figure 2D (column 3) and Figure 2E (column 3), FLAG-APH-2 was specifically detected in the c-Jun and JunB immunoprecipitates, respectively. Taken together, these results demonstrate that APH-2 dimerizes with endogenous c-Jun and JunB.

### The non-conventional bZIP domain of APH-2 is critical for binding c-Jun and JunB and stimulating their transcriptional activation

The leucine zipper motif of a conventional bZIP domain is a protein-protein interaction domain consisting of amphipathic α-helices that dimerize either as homodimers or heterodimers to form a coiled-coil. Despite the lack of a conventional bZIP domain, APH-2 is still able to interact with CREB and repress Tax2-dependant activation of HTLV-2 gene transcription [25].

**Figure 3:**
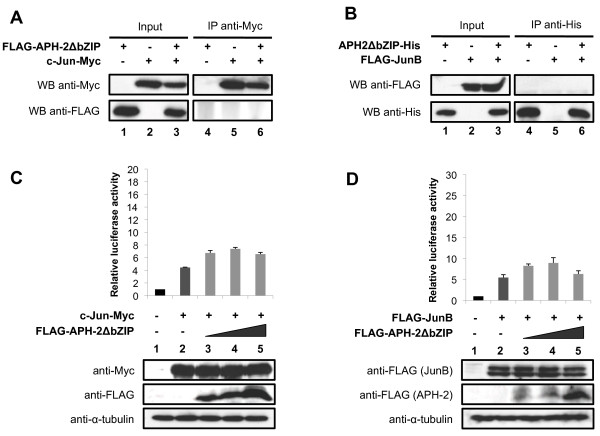
**APH-2ΔbZIP does not interact with c-Jun and JunB and fails to stimulate their transcriptional activity.** (**A** and **B**) APH-2ΔbZIP does not bind to c-Jun or JunB, respectively. Nuclear extracts from 293T cells transfected with the indicated vectors were prepared 48 hours post-transfection. Co-immunoprecipitations were then performed with Myc or His antibodies as indicated, followed by Western blotting with the indicated antibodies (WB). (**C** and **D**) APH-2ΔbZIP does not stimulate the transcriptional activity of c-Jun and JunB. The pAP-1 luciferase reporter construct was transiently co-transfected with the indicated expression vectors. Cells were lysed 48 hours post-transfection and processed for luciferase assays and Western blot analysis. The luciferase values represent an average of at least three independent experiments.

To assess whether the non-canonical bZIP domain of APH-2 is required for its interaction with c-Jun and JunB, we constructed a mutant of APH-2 that lacks the leucine zipper motif and named it APH-2ΔbZIP. Next, we performed co-immunoprecipitations with nuclear extracts from 293T cells overexpressing FLAG-APH-2ΔbZIP and/or c-Jun-Myc (Figure 3A) as well as APH-2ΔbZIP-His and/or FLAG-JunB (Figure 3B). Interestingly, neither c-Jun-Myc (Figure 3A, WB anti-FLAG, column 6) nor FLAG-JunB (Figure 3B, WB anti-FLAG, column 6) was able to co-immunoprecipitate with FLAG-APH-2ΔbZIP and APH-2ΔbZIP-His respectively, suggesting that APH-2ΔbZIP was no longer able to physically bind c-Jun and JunB.

Finally, to test whether the absence of the non-conventional bZIP domain could abolish the ability of APH-2 to activate c-Jun and JunB-mediated transactivation, we carried out luciferase assays. 293T were transfected with the AP-1-Luc reporter construct, c-Jun-Myc or FLAG-JunB as well as APH-2ΔbZIP expression vectors (Figure 3C and 3D, respectively, upper panels). The expression levels of the transfected proteins were verified by Western blot (Figure 3C and 3D, lower panels). As expected, APH-2ΔbZIP was unable to stimulate the transcriptional activity of c-Jun and JunB (Figure 3C, columns 3–5 and Figure 3D, columns 3–5, respectively). Similar experiments conducted with JunD show that even though APH-2ΔbZIP did not interact with JunD, the non-conventional bZIP domain is required for APH-2-mediated stimulation of JunD transactivation (Additional file 2A and 2B).

Altogether, these results demonstrate that APH-2 binds c-Jun and JunB via its non-conventional bZIP domain. Moreover, this domain is crucial for APH-2 ability to stimulate c-Jun and JunB-dependent AP-1 transcription.

### APH-2 interacts with Tax2B *in vitro* and *in vivo* and represses the ability of Tax2B to stimulate AP-1 transcription

**Figure 4:**
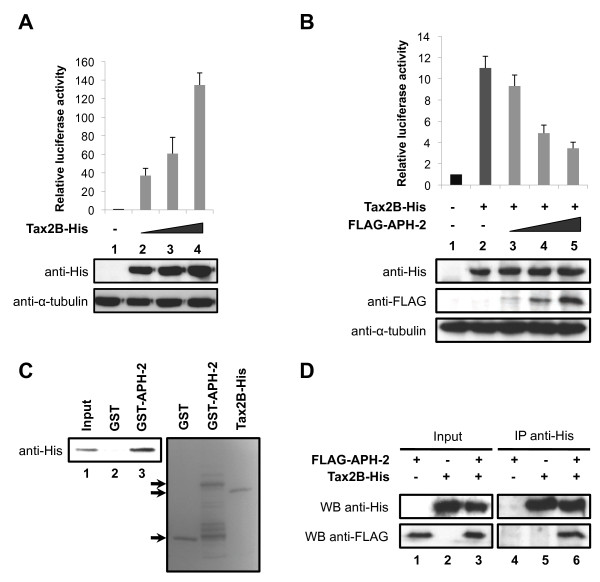
**APH-2 interacts with Tax2B and inhibits Tax2B-mediated activation of AP-1.** (**A** and **B**) APH-2 neutralizes the stimulatory effect of Tax2B on AP-1 activity. 293T cells were transiently co-transfected with the pAP-1 reporter vector as well as the indicated expression plasmids. Two days after transfection, the cells were lysed and luciferase activities were measured. The graphs show an average from at least three independent experiments. The proteins levels were analysed by Western blot using the indicated antibodies. (**C**) APH-2 interacts with Tax2B *in vitro*. Purified Tax2B-His was incubated with GST or GST-APH-2 and a pull-down was carried out. The precipitate was analyzed using His antibodies (left panel). The purified proteins were visualized by Coomassie blue staining (right panel). (**D**) APH-2 forms a complex with Tax2B *in vivo*. 293T cells were transiently transfected with the indicated expression plasmids. Nuclear extracts were prepared two days after transfection and subjected to immunoprecipitation with His antibodies. Proteins in the immunoprecipitates were analyzed by Western blot using the indicated antibodies (WB).

It has been previously reported that the HTLV-1 Tax oncoprotein activates AP-1-mediated transcription [18]. To examine whether HTLV-2 Tax2B was also able to affect AP-1 transcriptional activity, we carried out luciferase assays using lysates from cells transfected with the AP-1-Luc reporter construct together with increasing amounts of Tax2B expression vector (Figure 4A, upper panel). The amounts of Tax2B-His transfected were confirmed by Western blot (Figure 4A, lower panel). Our results show that, similar to Tax1, Tax2B stimulates AP-1 activity in a dose-dependent manner (Figure 4A, columns 2–4).

Taken together, our data show that both HTLV-2 proteins Tax2B and APH-2 are individually able to activate AP-1 transcription. In order to monitor AP-1 activity when Tax2B and APH-2 are co-expressed, we conducted luciferase assays on 293T cells co-transfected with the AP1-Luc reporter construct and Tax2B-His and/or FLAG-APH-2 expression vectors (Figure 4B, upper panel). The expression levels of Tax2B-His and FLAG-APH-2 were confirmed by Western blot analysis (Figure 4B, lower panel). We speculated that Tax2B and APH-2 effects on AP-1 activity are either additive or synergistic. Unexpectedly, our data demonstrate that APH-2 suppressed the activation of AP-1 transcription by Tax2B, indicating that APH-2 acts as a repressor of Tax2B-mediated transactivation (Figure 4B, columns 3–5).

We next investigated whether Tax2B and APH-2 interact *in vitro*. To address this issue, we performed GST pull-down assays with GST-APH-2 incubated with purified Tax2B-His. As illustrated in Figure 4C, GST-APH-2 binds Tax2B-His but not GST, indicating that APH-2 interacts directly with Tax2B (Figure 4C, columns 2 and 3).

To test whether APH-2 and Tax2B also interact *in vivo*, cells were transfected with Tax2B-His and/or FLAG-APH-2 and cellular lysates were subjected to co-immunoprecipitation assays with anti-His antibodies (Figure 4D). Our data demonstrate that FLAG-APH-2 was detected in the immunoprecipitates from cells co-transfected with FLAG-APH-2 and Tax2B-His (Figure 4D, WB anti-FLAG, column 6) but not FLAG-APH-2 or Tax2B-His alone (Figure 4D, WB anti-FLAG, columns 4 and 5).

As previously described, Tax2B is mainly distributed in the cytoplasm but can also be found in punctate nuclear structures whereas APH-2 displays a predominant nuclear localization [25,41]. To test whether the expression of Tax2B was able to alter APH-2 localization, we carried out immunofluorescence experiments. Interestingly, Tax2B expression was able to relocate APH-2 to the nuclear periphery (Additional file 3A).

Altogether, these results suggest that Tax2B-His and FLAG-APH-2 form a stable protein complex *in vitro* and *in vivo*.

### APH-2 and c-Jun/JunB interaction is independent of Tax2B

**Figure 5:**
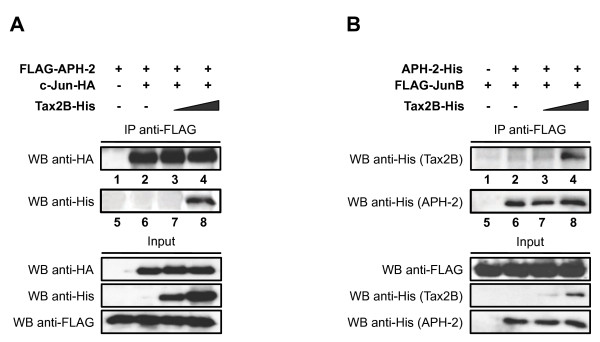
**Tax2B does not compete with c-Jun and JunB in their interaction with APH-2.** (**A** and **B**) Competition-binding assays were performed with nuclear extracts from 293T cells overexpressing the indicated tagged-proteins. Co-immunoprecipitations were carried out using FLAG antibodies and the co-immunoprecipitated proteins were detected by Western blot using the indicated antibodies (WB).

In the present study, we identified two members of the Jun family and the viral protein Tax2B as new APH-2 interaction partners. To investigate whether Tax2B and c-Jun/JunB compete for APH-2 interaction when the three protein partners are co-expressed, we performed immunoprecipitation assays (Figure 5A and 5B). To this end, nuclear extracts from cells expressing FLAG-APH-2, c-Jun-HA and increasing amounts of Tax2B-His were immunoprecipitated with FLAG antibodies. The presence of c-Jun-HA in the immunoprecipitates was then analyzed by Western blot using HA antibodies (Figure 5A). Results show that the relative amounts of c-Jun-HA detected in the immunoprecipitates were independent of Tax2B-His expression (Figure 5A, columns 2–4). Interestingly, Tax2B-His was also co-immunoprecipitated, suggesting that Tax2B is also part of the APH-2/c-Jun complex (Figure 5A, columns 7–8).

As a control, we tested whether c-Jun was able to affect Tax2B localization. The results obtained from our immunofluorescence experiments show that c-Jun was able to delocalize Tax2B from the cytoplasm to the nucleus (Additional file 3B).

Similar co-immunoprecipitation experiments were performed with nuclear extracts from cells transfected with APH-2-His and FLAG-JunB either alone or together with increasing amounts of Tax2B-His and immunoprecipitated with FLAG antibodies (Figure 5B). As expected, Tax2B was also part of the APH-2/JunB complex (Figure 5B, columns 3–4) but did not affect the interaction between APH-2 and JunB (Figure 5B, columns 6–8). Conversely, additional co-immunoprecipitations reveal that c-Jun and JunB did not have an effect on the interaction between APH-2 and Tax2B (Additional file 4A and 4B, respectively).

Overall our results strongly suggest that APH-2, Tax2B and c-Jun/JunB can form a ternary complex. Similarly, Tax2A also binds APH-2 but this interaction does not affect the association between APH-2 and c-Jun/JunB (Additional file 5A and 5B, respectively).

### The interaction between APH-2 and Tax2B neither involves the non-canonical bZIP domain nor the LXXLL domain of APH-2

**Figure 6:**
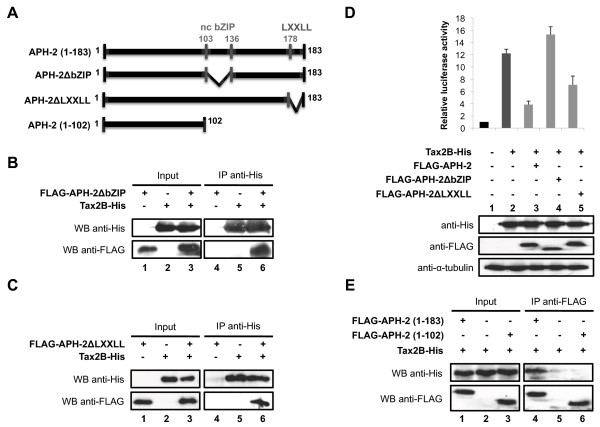
**The non-conventional bZIP domain and the LXXLL motif of APH-2 are not involved in its interaction with Tax2B.** (**A**) Schematic representation of the APH-2 mutants. (**B**, **C** and **E**) Mapping of the interaction between APH-2 and Tax2B. 293T cells were transiently transfected with the indicated expression plasmids. Cellular extracts were prepared and immunoprecipitated with the indicated antibodies (IP) 48 hours post-transfection. The presence of the proteins of interest in the immunoprecipitates was visualized by Western blotting using the indicated antibodies (WB). (**D**) The non-canonical bZIP domain of APH-2 is required to inhibit Tax2B-mediated AP-1 transcription. 293T cells were transfected with the pAP-1 luciferase construct and the indicated expression vectors. Luciferase and Renilla activities were measured 48 hours after transfection. The average of at least three different experiments is shown. The expression levels of the transfected proteins were analysed by Western blot using the indicated antibodies.

Thus far, we have demonstrated that there is no competition between Tax2B and the Jun family members for APH-2 binding (Figure 5). Interestingly, we also reported that APH-2 interacts with c-Jun and JunB through its non-canonical bZIP domain (Figure 3A and 3B). We then speculated that the interaction between APH-2 and Tax2B might not involve the non-canonical bZIP domain of APH-2. To confirm this hypothesis, co-immunoprecipitations of the FLAG-APH-2ΔbZIP mutant with Tax2B-His were determined using His antibodies followed by Western blot with FLAG antibodies to detect FLAG-APH-2ΔbZIP (Figure 6B). As predicted, FLAG-APH-2ΔbZIP was present in the immunoprecipitate, confirming that APH-2 and Tax2B interact *in vivo* but not through the non-conventional bZIP domain of APH-2 (Figure 6B, WB anti-FLAG, columns 4–6). Overall, these results reveal that c-Jun and JunB, but not Tax2B, interact with APH-2 though its non-conventional bZIP domain, which is consistent with our data showing that c-Jun/JunB and Tax2B do not compete for APH-2.

A recent study reported that the LXXLL motif of APH-2 is important for CREB binding and repression of Tax function on viral genes [42]. We therefore tested whether this motif was also involved in Tax2B binding and repression of Tax2B function on AP-1 transcription. To this aim, we generated a mutant of APH-2 that lacks the LXXLL motif and named it APH-2ΔLXXLL (Figure 6A). Co-immunoprecipitations were carried out with cellular extracts overexpressing FLAG-APH-2ΔLXXLL and/or Tax2B-His and immunoprecipitated with His antibodies (Figure 6C). Results revealed that FLAG antibodies were able to detect FLAG-APH-2ΔLXXLL in nuclear extracts overexpressing both proteins, thus suggesting that Tax2B can still interact with the APH-2ΔLXXLL mutant (Figure 6C, WB anti-FLAG, column 6).

Furthermore, we carried out luciferase assays to test the effects of these APH-2 mutants on Tax2B-mediated AP-1 transcription (Figure 6D, upper panel). 293T cells were transfected with the AP-1-Luc reporter construct, Tax2B and APH-2 full-length, ΔbZIP or ΔLXXLL. The expression levels of Tax2B-His and FLAG-APH-2 constructs were confirmed by Western blot analysis (Figure 6D, lower panel). Interestingly, even if the non-canonical bZIP domain of APH-2 is not required for its interaction with Tax2B, it appears crucial for Tax2B function on AP-1 transcription as APH-2-mediated repression of Tax2B function is completely inhibited when this domain is deleted (Figure 6D, column 4). Unlike ΔbZIP, the ΔLXXLL mutant was not able to abolish the ability of APH-2 to repress Tax2B-mediated transactivation of AP-1 (Figure 6D, column 5).

Thus far, we have demonstrated that the interaction between APH-2 and Tax2B does not involve the two main domains of APH-2: the bZIP domain (103–136) and the LXXLL domain (178–186). In order to further study the interaction between APH-2 and Tax2B, we constructed a FLAG-tagged mutant of APH-2 lacking the C-terminal part of the protein: APH-2 (1–102) (Figure 6A). We then performed co-immunoprecipitations with cellular extracts from 293T cells overexpressing Tax2B and FLAG-APH-2 full-length or N-terminal (1–102) in combination with Tax2B-His (Figure 6E). Our data reveal that in contrast with the full-length APH-2 (Figure 6E, WB anti-His, column 4), the N-terminal part of APH-2 (1–102) is unable to interact with Tax2B-His (Figure 6E, WB anti-His, column 6). Overall, these results suggest that, by default, APH-2 interacts with Tax2B through its C-terminal part (137–177).

### APH-2 and Tax2B finely regulate the activity of the collagenase promoter

**Figure 7:**
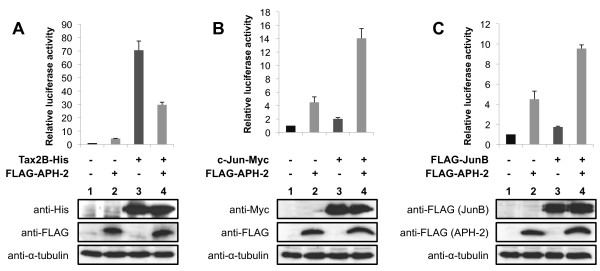
**APH-2 and Tax2B modulate the activity of the collagenase promoter.** 293T cells were transfected with the reporter plasmid pCollagenase-Luc and the indicated expression vectors. Luciferase and Renilla activities were measured 2 days after transfection. The average of at least three different experiments is shown. The expression levels of Tax2B-His, c-Jun-Myc, FLAG-JunB and FLAG-APH-2 were visualized by Western blot using the indicated antibodies. (**A**) APH-2 suppresses Tax2B-induced collagenase transcription (**B** and **C**) APH-2 activates c-Jun and JunB-mediated transactivation of the collagenase promoter, respectively.

Our report highlights the role of the viral proteins APH-2 and Tax2B in the regulation of AP-1 activity using a minimal promoter harbouring AP-1 binding sites as a model. To examine the physiological relevance of these findings, we investigated the effect of APH-2 and Tax2B on the human collagenase promoter, which contains a TRE [43,44]. We performed luciferase assays using a construct containing the luciferase gene driven by the human collagenase promoter (Figure 7). The reporter plasmid along with Tax2B, c-Jun or JunB expression plasmids were co-transfected in the absence or presence of APH-2, and the cell lysates were processed for luciferase assays (Figures 7A, 7B and 7C, respectively, upper panels) and Western blot (Figures 7A, 7B and 7C, respectively, lower panels). We observed that APH-2 and Tax2B overexpressed separately activated the collagenase promoter as predicted by our study (Figure 7A, columns 2 and 3). In the cells co-expressing both viral proteins, APH-2 acted as an inhibitor by suppressing Tax2B ability to induce collagenase transcription (Figure 7A, column 4). The regulation of collagenase gene transcription has been well documented and both c-Jun and JunB were reported to activate the collagenase promoter [45,46]. We were able to confirm these findings in the context of our experiments as c-Jun and JunB stimulated the activity of the collagenase promoter by approximately 2 fold (Figures 7B and 3C, columns 3). Co-expression of APH-2 resulted in a stimulation of c-Jun and JunB-mediated transactivation of about 14 and 9-fold activation relative to the basal level, respectively (Figure 7B and 7C, columns 4).

These findings demonstrate that APH-2 acts synergistically with c-Jun and JunB to activate transcription of the collagenase promoter. Taken together our data suggest that the collagenase promoter well illustrates how APH-2 and Tax2B regulate AP-1 activity.

## Discussion

HTLV-1 and HTLV-2 differ both in their specific epidemiologic and pathogenic properties. In contrast to HTLV-1 that causes ATL and HAM/TSP, HTLV-2 has not been clearly linked to any disease but has been associated with lymphocyte proliferation and high platelet counts as well as rare cases of chronic neuromyelopathy [7-9]. This suggests that HTLV-2 fails to promote a critical stage of leukemogenesis and neurologic disease development.

The distinct clinical manifestations of HTLV-1 and HTLV-2 can be attributed in part to distinct biological functions of Tax1 and Tax2 [47]. For instance, the transforming potential of Tax1 is higher than that of Tax2 mainly due to the fact that, unlike Tax1, Tax2 cannot activate the non-canonical NF-κB pathway because of its inability to interact with NF-κB2/p100 [48,49].

Although Tax plays a pivotal role in HTLV-associated transformation of T-cells, the *tax* gene is frequently genetically and epigenetically inactivated in ATL cells, suggesting that Tax is not required for the maintenance of the leukemic stage in ATL [50,51]. Recent studies showed that transcription from the 3’ LTR of the HTLV genomes governs the expression of antisense regulatory proteins [24-26]. HBZ, the antisense protein encoded by HTLV-1, is a bZIP factor that is consistently expressed in ATL cells and plays a key role in the malignant proliferation in ATL [52]. HBZ interacts with numerous cellular transcription factors of the bZIP family and modulates their transcriptional activity. Notably, by interacting with c-Jun, JunB and JunD, HBZ highly influences AP-1 transcription [33,34].

In the current study, we reveal that the antisense protein of HTLV-2 (APH-2) also regulates AP-1 activity. However, we postulate that HBZ and APH-2 display opposite effects on AP-1 basal transcription. While HBZ inhibits AP-1-mediated transcription, APH-2 acts as an activator of AP-1 basal activity. Moreover, whereas HBZ has been described as an inhibitor of c-Jun and JunB-mediated transcription [33,38], we demonstrated that APH-2 enhances the transcriptional activity of c-Jun and JunB on AP-1 binding sites. As AP-1 controls numerous biological processes critical in virus replication such as oncogenic transformation, cell proliferation, differentiation and apoptosis, we hypothesize that this divergence in AP-1 transcription regulation might explain, in part, the differences between HTLV-1 and HTLV-2 pathogenesis. A recent study already excluded the involvement of APH-2 in lymphocyte proliferation [40], but further investigation is needed to establish the role of APH-2 in other AP-1-associated biological functions.

We focused our studies on elucidating the molecular mechanisms involved in the regulation of AP-1 activity by APH-2 and demonstrated that APH-2 physically interacted with c-Jun and JunB through its non-conventional bZIP domain. HBZ harbours a bZIP domain that is involved in numerous biological functions and especially in its interaction with c-Jun and JunB [33]. Interestingly, despite the lack of a consensus bZIP domain, APH-2 was still able to interact with c-Jun and JunB and therefore still share some similarities with HBZ. Using a reporter assay, we established that this non-canonical bZIP domain is critical for APH-2 to enhance c-Jun and JunB transcriptional activity on AP-1 transcription. These findings confirm that the physical interaction between APH-2 and c-Jun/JunB is essential for APH-2 to regulate c-Jun and JunB-mediated AP-1 transcription.

Interestingly, as previously described for HBZ [34], APH-2 cooperates with JunD through its non-canonical bZIP domain to stimulate the AP-1 activity. However, our results show that APH-2 and JunD are not able to form a stable complex *in vivo*. We postulate that APH-2 and JunD might involve a different mechanism of action than APH-2 and c-Jun/JunB to stimulate AP-1 transcription. Further investigation is needed to better understand how APH-2 indirectly stimulates JunD transcriptional activity on AP-1 transcription.

The development of ATL in HTLV-1 infected patients has been associated with the deregulation of cellular gene transcription. Among all HTLV-1 proteins, Tax1 is known to play a critical role in ATL development by disrupting major cellular signal transduction pathways such as NF-κB and AP-1 [18,47]. In the present study, we reveal that HTLV-2 Tax enhances AP-1 transcription. However, when Tax2B and APH-2 are co-expressed, they interact directly and APH-2 impairs the ability of Tax2B to activate AP-1 transcription. These functional effects could be explained by the fact that Tax2B can relocate APH-2 to the nuclear periphery, a mechanism that could prevent APH-2 from activating AP-1 transcription. Interestingly, similar functional effects have been described on the HTLV-2 promoter [25], but further experiments are required to investigate whether Tax2B and APH-2 are using a common molecular mechanism to regulate HTLV-2 and AP-1 transcription.

Here, we report that APH-2, Tax2B and Jun can form a ternary complex, as there is no competition between Tax2B and c-Jun/JunB for APH-2 binding. Indeed, unlike c-Jun/JunB, APH-2 and Tax2B, interaction does not involve the non-canonical bZIP domain of APH-2. However, this domain, and consequently the interaction between APH-2 and c-Jun/JunB, is crucial to repress Tax function on AP-1 transcription.

According to a recent study, the LXXLL motif of APH-2 is involved in its interaction with CREB and in the repression of Tax function on viral genes [42]. Surprisingly, we demonstrated that this motif is not essential for APH-2 repressive function on Tax2B-mediated AP-1 transcription and is not involved in Tax2B binding.

The human collagenase promoter is an extensively studied AP-1 responsive promoter [43,44]. Here we used the collagenase promoter as an example to illustrate AP-1 transcription regulation by the two HTLV-2 proteins APH-2 and Tax2B. We established that, according to our model, APH-2 stimulates c-Jun and JunB-mediated transactivation. Moreover, APH-2 or Tax2B expressed individually highly enhance AP-1 transcription whereas co-expression of both viral proteins results in a suppression of Tax2B-mediated transactivation by APH-2. We also speculate that the molecular mechanism by which APH-2 and Tax2B regulate the collagenase transcription could be common to numerous AP-1 target genes.

A recent study reported that both APH-2 and Tax2 mRNA expression are correlated with HTLV-2 proviral loads (PVL). However, whereas *APH2* was expressed *in vivo* in the majority of HTLV-2 carriers, *Tax* expression was not detected among HTLV-2 carriers with low PVL [40]. With the intention of correlating this study with our findings, we speculate that in HTLV-2 carriers with low PVL, only APH-2 is expressed and consequently, AP-1 activity is stimulated. However, in HTLV-2 carriers with high PVL, APH-2 and Tax2 are co-expressed and AP-1 activity is down-regulated, suggesting that APH-2 might balance the transactivation activity of Tax2B on HTLV-2 and AP-1 transcription in order to silence the virus and allow it to escape host immune responses.

## Conclusions

The present study is the first to demonstrate that HTLV-2 deregulates AP-1 activity. Moreover, together with a previous report [25], we confirm that APH-2 and Tax2B act as viral transcription factors that subtly regulate HTLV-2 and AP-1 transcription to possibly help the virus to replicate and counteract host immune responses. Moreover, in accordance with previous studies [33,34,38], we report that APH-2 and HBZ display similar repressive effects on AP-1-mediated transcription in the presence of Tax but have opposite effects in its absence. These findings highlight the fact that APH-2 and HBZ have distinct biological properties that may contribute to the differential pathogenic potential of HTLV-1 and HTLV-2.

## Methods

### Cells and plasmids

The 293T cells (obtained from the ATCC) were cultured under standard tissue culture conditions. The pAP1-Luc and pRL-TK-Renilla plasmids are commercially available at Stratagene and Promega, respectively. The pCAGGS-Tax2B-His was previously described [53]. The pFLAG-JunB and pFLAG-JunD were generous gifts from Dr. Rong Li (Stowers Institute for Medical Research, Kansas City, USA). The p-c-Jun-HA, pCollagenase-Luc and p-c-Jun-Myc were kindly provided by Dr. Anna Maria Musti (University of Würzburg, Germany), Dr. Sagar Ghosh (University of Texas, San Antonio, USA) and Dr. Kunitada Shimotohno (Chiba Institute of Technology, Japan), respectively. The pGFP-APH-2 was obtained by cloning the APH-2 cDNA into the pEGFP (Invitrogen) using standard techniques. The pFLAG-APH-2 and pFLAG-HBZ were generated by cloning the APH-2 or HBZ cDNAs as a HindIII/EcoRI fragment obtained from pcDNA-APH-2-Myc-His or pcDNA-HBZ-Myc-His (kindly provided by Dr. Jean-Michel Mesnard, Université de Montpellier, France) into pFLAG-CMV (Sigma-Aldrich). The pFLAG-APH-2ΔbZIP and pAPH-2ΔbZIP-His mutants were generated by site-directed mutagenesis (Phusion® Site-Directed Mutagenesis kit, Thermo Scientific) using pFLAG-APH-2 or pcDNA-APH-2-Myc-His as a template and the following primers: 5’-TATACACTCCAACTGCTGATGCCTTTC-3’ and 5’-GAGGAACTATTTGAGGCAATTATTCAG-3’. The pFLAG-APH-2ΔLXXLL and pFLAG-APH-2 (1–102) mutants were also constructed by site-directed mutagenesis (Phusion® Site-Directed Mutagenesis kit, Thermo Scientific) using pFLAG-APH-2 as a template and the primers: 5’-TAAGAATTCATCGATAGATCTGATATCGGT-3’ and 5’- CTTCTGCAGCAAATCCCCATGGTT-3’ for pFLAG-APH-2ΔLXXLL and 5’-TAAGAATTCATCGATAGATCTGATATCGGT-3’ and 5’- TATACACTCCAACTGCTGATGCCTTTC-3’ for pFLAG-APH-2 (1–102). To obtain the GST-APH-2 construct, we generated an APH-2 PCR product using the pcDNA-APH-2-Myc-His as a template. The APH-2 PCR product was then digested with BamHI/EcoRI and cloned into the pGEX-2 T (GE Healthcare).

### Luciferase assays

293T cells were transiently transfected with the pAP1-Luc or pcollagenase reporter plasmids, different combinations of expression vectors and the pRL-TK-Renilla vector as an internal control using Lipofectamine™ 2000 (Invitrogen). DNA amounts were normalized across samples using the respective empty vectors. Cells were harvested 48 hours post-transfection and processed for luciferase assays (Dual Luciferase® Reporter Assay System, Promega) or Western blot.

### Co-immunoprecipitations

293T cells were transiently transfected with different combinations of expression vectors using Lipofectamine™ 2000 (Invitrogen). DNA amounts were normalized across samples using the respective empty vectors. Protein extracts were prepared 48 hours after transfection (NE-PER® Nuclear and Cytoplasmic Extraction Reagents, Thermo Scientific) and 400ug of protein extracts were immunoprecipitated with 5ug of anti-FLAG® M2 (Sigma-Aldrich, F3165), anti-Myc (Invitrogen, R950), anti-6xHis (Clontech, 631212), anti-c-Jun (Abcam ab31419), anti-JunB (Abcam ab31421) or rabbit IgG (Millipore) overnight at 4°C. Protein A/G PLUS-Agarose beads (Santa Cruz Biotechnology) were then added to the samples and incubated for 2 hours at 4°C. Beads were washed 3 times with IPLS buffer (50 mM Tris–HCl pH7.5, 120 mM NaCl, 0.5 mM EDTA, 0.5% NP-40), 3 times with IPHS buffer (50 mM Tris–HCl pH7.5, 400 mM NaCl, 0.5 mM EDTA, 0.5% NP-40) and twice with IPLS buffer. Samples were then subjected to Western blot.

### Western blotting

Cell lysates were subjected to SDS-PAGE and analyzed by Western blot using standard procedures. Membranes were probed using the SNAP i.d. system (Millipore). The antibodies used for Western blot are as follows: anti-FLAG® M2 (Sigma-Aldrich, F3165), anti-α-tubulin (Abcam, Ab7291), anti-Myc (Invitrogen, R950), anti-6xHis (Clontech, 631212) and anti-HA (Invitrogen, 32–6700).

### GST pull-down

Purified GST or GST-APH-2 fusion proteins were immobilized on Glutathione Sepharose™ 4 Fast Flow beads (GE Healthcare) and incubated with purified Tax2B-His overnight at 4°C. After extensive washing with GST Wash Buffer (0.5% Triton® X-100 in PBS supplemented with protease inhibitors), bound proteins were eluted using GST Elution Buffer (50 mM Tris–HCl pH8.0, 10 mM reduced glutathione) and separated by Western blot. Tax2B-His was detected with anti-6xHis antibodies (Clontech, 631212).

### Immunofluorescence

COS-7 cells were plated onto chamber slides and co-transfected with the indicated expression vectors using FuGENE® HD (Promega). One day post-transfection, cells were washed with PBS, fixed with 4% paraformaldehyde for 15 minutes at room temperature, permeabilized with 0.2% Tween/PBS for 10 minutes at room temperature and incubated with a blocking reagent (TSA kit, Molecular Probes) for 1 hour at room temperature. Tax2B was detected with an anti-Tax2B antibody (Fusion Antibodies) followed by anti-mouse IgG-HRP and Alexa Fluor® 594 tyramide staining (Molecular Probes). c-Jun-Myc was detected using an anti-Myc-HRP antibody (Invitrogen, R951) followed by Alexa Fluor® 488 tyramide staining (Molecular Probes). DAPI (Sigma-Aldrich) was used to stain the nuclei. Slides were mounted using the ProLong® Gold Antifade reagent (Invitrogen). Images were acquired with a Zeiss Axio Imager microscope.

## Competing interests

The authors declare that they have no competing interests.

## Authors’ contributions

Conceived and design the study: CM, WWH, NS. Performed the experiments: CM. Carried out the immunofluorescence experiments: AMC. Contributed to cloning: TNB. Wrote the paper: CM. Helped to draft the manuscript: WWH, NS. All authors read and approved the final manuscript.

## Supplementary Material

Additional file 1 **APH-2 associates with c-Jun and JunB but not JunD.** 293T cells were transiently transfected with the indicated expression plasmids. Two days after transfection, nuclear extracts were immunoprecipitated with the indicated antibodies (IP). The presence of proteins of interest in the immunoprecipitates was visualized by Western blot using the indicated antibodies (WB). (**A**) APH-2 interacts with c-Jun. (**B**) APH-2 binds JunB. (**C**) APH-2 does not interact with JunD. Click here for file

Additional file 2 **APH-2ΔbZIP fails to stimulate JunD-mediated transcriptional activity.** (**A**) APH-2ΔbZIP does not bind to JunD. Nuclear extracts from 293T cells transfected with the indicated vectors were prepared 48 hours post-transfection. Co-immunoprecipitations were then performed with His antibodies, followed by Western blotting with the indicated antibodies (WB). (**B**) APH-2ΔbZIP does not stimulate the transcriptional activity of JunD. The pAP-1 luciferase reporter construct was transiently co-transfected with the indicated expression vectors. Cells were lysed 48 hours post-transfection and processed for luciferase assays and Western blot analysis. The luciferase values represent an average of at least three independent experiments. Click here for file

Additional file 3 **Subcellular localization of APH**-**2 and c**-**Jun in the presence of Tax2B.** COS-7 cells were transfected with the indicated plasmids. Cells were fixed and permealized 24 hours post-transfection. The proteins of interest were immunodetected and stained as indicated. Nuclei were stained with DAPI. Immunofluorescence images were obtained with a Zeiss Axio Imager microscope. Representative images of the entire cell population are shown. (**A**) Tax2B relocates APH-2 to the nuclear periphery. (**B**) c-Jun relocates Tax2B in the cell nuclei. Click here for file

Additional file 4 **c-Jun and JunB do not compete with Tax2B in its interaction with APH-2.** Competition-binding assays were performed with nuclear extracts from 293 T cells overexpressing the indicated tagged-proteins. Co-immunoprecipitations were carried out using the indicated antibodies and the co-immunoprecipitated proteins were detected by Western blot using the indicated antibodies (WB). (**A** and **B**) c-Jun and JunB do not affect the interaction between APH-2 and Tax2B. Click here for file

Additional file 5 **APH-2 and c-Jun/JunB interaction is independent of Tax2A.** (**A** and **B**) Tax2A does not affect the interaction between APH-2 and c-Jun/JunB. Competition-binding assays were performed with nuclear extracts from 293 T cells overexpressing the indicated tagged-proteins. Co-immunoprecipitations were carried out using FLAG antibodies and the co-immunoprecipitated proteins were detected by Western blot using the indicated antibodies. Click here for file

## References

[B1] Calattini S Chevalier SA Duprez R Bassot S Froment A Mahieux R Gessain A Discovery of a new human T-cell lymphotropic virus (HTLV-3) in Central Africa Retrovirology 2005 2 30 10.1186/1742-4690-2-30 15882466 PMC1142341

[B2] Kalyanaraman VS Sarngadharan MG Robert-Guroff M Miyoshi I Golde D Gallo RC A new subtype of human T-cell leukemia virus (HTLV-II) associated with a T-cell variant of hairy cell leukemia Science 1982 218 571 573 10.1126/science.6981847 6981847

[B3] Poiesz BJ Ruscetti FW Reitz MS Kalyanaraman VS Gallo RC Isolation of a new type C retrovirus (HTLV) in primary uncultured cells of a patient with Sezary T-cell leukaemia Nature 1981 294 268 271 10.1038/294268a0 6272125

[B4] Wolfe ND Heneine W Carr JK Garcia AD Shanmugam V Tamoufe U Torimiro JN Prosser AT Lebreton M Mpoudi-Ngole E Emergence of unique primate T-lymphotropic viruses among central African bushmeat hunters Proc Natl Acad Sci U S A 2005 102 7994 7999 10.1073/pnas.0501734102 15911757 PMC1142377

[B5] Uchiyama T Yodoi J Sagawa K Takatsuki K Uchino H Adult T-cell leukemia: clinical and hematologic features of 16 cases Blood 1977 50 481 492 301762

[B6] Iwasaki Y Pathology of chronic myelopathy associated with HTLV-I infection (HAM/TSP) J Neurol Sci 1990 96 103 123 10.1016/0022-510X(90)90060-Z 2351985

[B7] Bartman MT Kaidarova Z Hirschkorn D Sacher RA Fridey J Garratty G Gibble J Smith JW Newman B Yeo AE Murphy EL Long-term increases in lymphocytes and platelets in human T-lymphotropic virus type II infection Blood 2008 112 3995 4002 10.1182/blood-2008-05-155960 18755983 PMC2581993

[B8] Hall WW Ishak R Zhu SW Novoa P Eiraku N Takahashi H Ferreira Mda C Azevedo V Ishak MO Ferreira Oda C Human T lymphotropic virus type II (HTLV-II): epidemiology, molecular properties, and clinical features of infection J Acquir Immune Defic Syndr Hum Retrovirol 1996 13 Suppl 1 S204 S214 8797725 10.1097/00042560-199600001-00031

[B9] Roucoux DF Murphy EL The epidemiology and disease outcomes of human T-lymphotropic virus type II AIDS Rev 2004 6 144 154 15595431

[B10] Ross TM Narayan M Fang ZY Minella AC Green PL Human T-cell leukemia virus type 2 tax mutants that selectively abrogate NFkappaB or CREB/ATF activation fail to transform primary human T cells J Virol 2000 74 2655 2662 10.1128/JVI.74.6.2655-2662.2000 10684280 PMC111754

[B11] Sun SC Ballard DW Persistent activation of NF-kappaB by the tax transforming protein of HTLV-1: hijacking cellular IkappaB kinases Oncogene 1999 18 6948 6958 10.1038/sj.onc.1203220 10602469

[B12] Zhao LJ Giam CZ Interaction of the human T-cell lymphotrophic virus type I (HTLV-I) transcriptional activator Tax with cellular factors that bind specifically to the 21-base-pair repeats in the HTLV-I enhancer Proc Natl Acad Sci U S A 1991 88 11445 11449 10.1073/pnas.88.24.11445 1763059 PMC53152

[B13] Zhao LJ Giam CZ Human T-cell lymphotropic virus type I (HTLV-I) transcriptional activator, Tax, enhances CREB binding to HTLV-I 21-base-pair repeats by protein-protein interaction Proc Natl Acad Sci U S A 1992 89 7070 7074 10.1073/pnas.89.15.7070 1386673 PMC49647

[B14] Suzuki T Fujisawa JI Toita M Yoshida M The trans-activator tax of human T-cell leukemia virus type 1 (HTLV-1) interacts with cAMP-responsive element (CRE) binding and CRE modulator proteins that bind to the 21-base-pair enhancer of HTLV-1 Proc Natl Acad Sci U S A 1993 90 610 614 10.1073/pnas.90.2.610 8421695 PMC45713

[B15] Goren I Semmes OJ Jeang KT Moelling K The amino terminus of Tax is required for interaction with the cyclic AMP response element binding protein J Virol 1995 69 5806 5811 7637025 10.1128/jvi.69.9.5806-5811.1995 PMC189446

[B16] Paca-Uccaralertkun S Zhao LJ Adya N Cross JV Cullen BR Boros IM Giam CZ In vitro selection of DNA elements highly responsive to the human T-cell lymphotropic virus type I transcriptional activator Tax. Mol Cell Biol 1994 14 456 462 10.1128/mcb.14.1.456-462.1994 PMC358395 8264613

[B17] Fujii M Tsuchiya H Chuhjo T Akizawa T Seiki M Interaction of HTLV-1 Tax1 with p67SRF causes the aberrant induction of cellular immediate early genes through CArG boxes Genes Dev 1992 6 2066 2076 10.1101/gad.6.11.2066 1427072

[B18] Iwai K Mori N Oie M Yamamoto N Fujii M Human T-cell leukemia virus type 1 tax protein activates transcription through AP-1 site by inducing DNA binding activity in T cells Virology 2001 279 38 46 10.1006/viro.2000.0669 11145887

[B19] Mori N Fujii M Iwai K Ikeda S Yamasaki Y Hata T Yamada Y Tanaka Y Tomonaga M Yamamoto N Constitutive activation of transcription factor AP-1 in primary adult T-cell leukemia cells Blood 2000 95 3915 3921 10845928

[B20] Fujii M Sassone-Corsi P Verma IM c-fos promoter trans-activation by the tax1 protein of human T-cell leukemia virus type I Proc Natl Acad Sci U S A 1988 85 8526 8530 10.1073/pnas.85.22.8526 2847164 PMC282491

[B21] Fujii M Tsuchiya H Seiki M HTLV-1 Tax has distinct but overlapping domains for transcriptional activation and for enhancer specificity Oncogene 1991 6 2349 2352 1766679

[B22] Landschulz WH Johnson PF McKnight SL The leucine zipper: a hypothetical structure common to a new class of DNA binding proteins Science 1988 240 1759 1764 10.1126/science.3289117 3289117

[B23] Shaulian E Karin M AP-1 in cell proliferation and survival Oncogene 2001 20 2390 2400 10.1038/sj.onc.1204383 11402335

[B24] Gaudray G Gachon F Basbous J Biard-Piechaczyk M Devaux C Mesnard JM The complementary strand of the human T-cell leukemia virus type 1 RNA genome encodes a bZIP transcription factor that down-regulates viral transcription J Virol 2002 76 12813 12822 10.1128/JVI.76.24.12813-12822.2002 12438606 PMC136662

[B25] Halin M Douceron E Clerc I Journo C Ko NL Landry S Murphy EL Gessain A Lemasson I Mesnard JM Human T-cell leukemia virus type 2 produces a spliced antisense transcript encoding a protein that lacks a classic bZIP domain but still inhibits Tax2-mediated transcription Blood 2009 114 2427 2438 10.1182/blood-2008-09-179879 19602711 PMC2746472

[B26] Larocque E Halin M Landry S Marriott SJ Switzer WM Barbeau B Human T-Cell Lymphotropic Virus Type 3 (HTLV-3)- and HTLV-4-Derived Antisense Transcripts Encode Proteins with Similar Tax-Inhibiting Functions but Distinct Subcellular Localization J Virol 2011 85 12673 12685 10.1128/JVI.05296-11 21917984 PMC3209360

[B27] Satou Y Yasunaga J Yoshida M Matsuoka M HTLV-I basic leucine zipper factor gene mRNA supports proliferation of adult T cell leukemia cells Proc Natl Acad Sci U S A 2006 103 720 725 10.1073/pnas.0507631103 16407133 PMC1334651

[B28] Saito M Matsuzaki T Satou Y Yasunaga J Saito K Arimura K Matsuoka M Ohara Y In vivo expression of the HBZ gene of HTLV-1 correlates with proviral load, inflammatory markers and disease severity in HTLV-1 associated myelopathy/tropical spastic paraparesis (HAM/TSP) Retrovirology 2009 6 19 10.1186/1742-4690-6-19 19228429 PMC2653460

[B29] Arnold J Zimmerman B Li M Lairmore MD Green PL Human T-cell leukemia virus type-1 antisense-encoded gene, Hbz, promotes T-lymphocyte proliferation Blood 2008 112 3788 3797 10.1182/blood-2008-04-154286 18689544 PMC2572803

[B30] Lemasson I Lewis MR Polakowski N Hivin P Cavanagh MH Thebault S Barbeau B Nyborg JK Mesnard JM Human T-cell leukemia virus type 1 (HTLV-1) bZIP protein interacts with the cellular transcription factor CREB to inhibit HTLV-1 transcription J Virol 2007 81 1543 1553 10.1128/JVI.00480-06 17151132 PMC1797566

[B31] Hagiya K Yasunaga J Satou Y Ohshima K Matsuoka M ATF3, an HTLV-1 bZip factor binding protein, promotes proliferation of adult T-cell leukemia cells Retrovirology 2011 8 19 10.1186/1742-4690-8-19 21414204 PMC3068935

[B32] Zhao T Yasunaga J Satou Y Nakao M Takahashi M Fujii M Matsuoka M Human T-cell leukemia virus type 1 bZIP factor selectively suppresses the classical pathway of NF-kappaB Blood 2009 113 2755 2764 10.1182/blood-2008-06-161729 19064727

[B33] Basbous J Arpin C Gaudray G Piechaczyk M Devaux C Mesnard JM The HBZ factor of human T-cell leukemia virus type I dimerizes with transcription factors JunB and c-Jun and modulates their transcriptional activity J Biol Chem 2003 278 43620 43627 10.1074/jbc.M307275200 12937177

[B34] Thebault S Basbous J Hivin P Devaux C Mesnard JM HBZ interacts with JunD and stimulates its transcriptional activity FEBS Lett 2004 562 165 170 10.1016/S0014-5793(04)00225-X 15044019

[B35] Barbeau B Mesnard JM Does the HBZ gene represent a new potential target for the treatment of adult T-cell leukemia? Int Rev Immunol 2007 26 283 304 10.1080/08830180701690843 18027202

[B36] Clerc I Hivin P Rubbo PA Lemasson I Barbeau B Mesnard JM Propensity for HBZ-SP1 isoform of HTLV-I to inhibit c-Jun activity correlates with sequestration of c-Jun into nuclear bodies rather than inhibition of its DNA-binding activity Virology 2009 391 195 202 10.1016/j.virol.2009.06.027 19595408

[B37] Hivin P Basbous J Raymond F Henaff D Arpin-Andre C Robert-Hebmann V Barbeau B Mesnard JM The HBZ-SP1 isoform of human T-cell leukemia virus type I represses JunB activity by sequestration into nuclear bodies Retrovirology 2007 4 14 10.1186/1742-4690-4-14 17306025 PMC1805765

[B38] Matsumoto J Ohshima T Isono O Shimotohno K HTLV-1 HBZ suppresses AP-1 activity by impairing both the DNA-binding ability and the stability of c-Jun protein Oncogene 2005 24 1001 1010 10.1038/sj.onc.1208297 15592508

[B39] Kuhlmann AS Villaudy J Gazzolo L Castellazzi M Mesnard JM Duc Dodon M HTLV-1 HBZ cooperates with JunD to enhance transcription of the human telomerase reverse transcriptase gene (hTERT) Retrovirology 2007 4 92 10.1186/1742-4690-4-92 18078517 PMC2235888

[B40] Douceron E Kaidarova Z Miyazato P Matsuoka M Murphy EL Mahieux R HTLV-2 APH-2 expression is correlated with proviral load but APH-2 does not promote lymphocytosis J Infect Dis 2012 205 82 86 10.1093/infdis/jir708 22065675 PMC3242747

[B41] Bertazzoni U Turci M Avesani F Di Gennaro G Bidoia C Romanelli MG Intracellular localization and cellular factors interaction of HTLV-1 and HTLV-2 Tax proteins: similarities and functional differences Viruses 2011 3 541 560 10.3390/v3050541 21994745 PMC3185761

[B42] Yin H Kannian P Dissinger N Haines R Niewiesk S Green PL HTLV-2 APH-2 is dispensable for in vitro immortalization, but functions to repress early viral replication in vivo J Virol 2012 86 8412 8421 10.1128/JVI.00717-12 22623800 PMC3421770

[B43] Angel P Imagawa M Chiu R Stein B Imbra RJ Rahmsdorf HJ Jonat C Herrlich P Karin M Phorbol ester-inducible genes contain a common cis element recognized by a TPA-modulated trans-acting factor Cell 1987 49 729 739 10.1016/0092-8674(87)90611-8 3034432

[B44] Lee W Mitchell P Tjian R Purified transcription factor AP-1 interacts with TPA-inducible enhancer elements Cell 1987 49 741 752 10.1016/0092-8674(87)90612-X 3034433

[B45] Angel P Karin M Specific members of the Jun protein family regulate collagenase expression in response to various extracellular stimuli Matrix Suppl 1992 1 156 164 1336108

[B46] Westermarck J Lohi J Keski-Oja J Kahari VM Okadaic acid-elicited transcriptional activation of collagenase gene expression in HT-1080 fibrosarcoma cells is mediated by JunB Cell Growth Differ 1994 5 1205 1213 7848922

[B47] Higuchi M Fujii M Distinct functions of HTLV-1 Tax1 from HTLV-2 Tax2 contribute key roles to viral pathogenesis Retrovirology 2009 6 117 10.1186/1742-4690-6-117 20017952 PMC2806368

[B48] Higuchi M Tsubata C Kondo R Yoshida S Takahashi M Oie M Tanaka Y Mahieux R Matsuoka M Fujii M Cooperation of NF-kappaB2/p100 activation and the PDZ domain binding motif signal in human T-cell leukemia virus type 1 (HTLV-1) Tax1 but not HTLV-2 Tax2 is crucial for interleukin-2-independent growth transformation of a T-cell line J Virol 2007 81 1900 11907 10.1128/JVI.00532-07 PMC2168800 17715223

[B49] Shoji T Higuchi M Kondo R Takahashi M Oie M Tanaka Y Aoyagi Y Fujii M Identification of a novel motif responsible for the distinctive transforming activity of human T-cell leukemia virus (HTLV) type 1 Tax1 protein from HTLV-2 Tax2 Retrovirology 2009 6 83 10.1186/1742-4690-6-83 19761585 PMC2754985

[B50] Furukawa Y Kubota R Tara M Izumo S Osame M Existence of escape mutant in HTLV-I tax during the development of adult T-cell leukemia Blood 2001 97 987 993 10.1182/blood.V97.4.987 11159527

[B51] Takeda S Maeda M Morikawa S Taniguchi Y Yasunaga J Nosaka K Tanaka Y Matsuoka M Genetic and epigenetic inactivation of tax gene in adult T-cell leukemia cells Int J Cancer 2004 109 559 567 10.1002/ijc.20007 14991578

[B52] Matsuoka M Green PL The HBZ gene, a key player in HTLV-1 pathogenesis Retrovirology 2009 6 71 10.1186/1742-4690-6-71 19650892 PMC2731725

[B53] Sheehy N Lillis L Watters K Lewis M Gautier V Hall W Functional analysis of human T lymphotropic virus type 2 Tax proteins Retrovirology 2006 3 20 10.1186/1742-4690-3-20 16551350 PMC1462996

