# Supplementary figures and images for: Interplay between the HTLV-2 Tax and APH-2 proteins in the regulation of the AP-1 pathway
Source: Retrovirology. 2012 Dec 3;9:98. doi: 10.1186/1742-4690-9-98 (PMC3531308; doi:10.1186/1742-4690-9-98)

Additional File 1

**A**

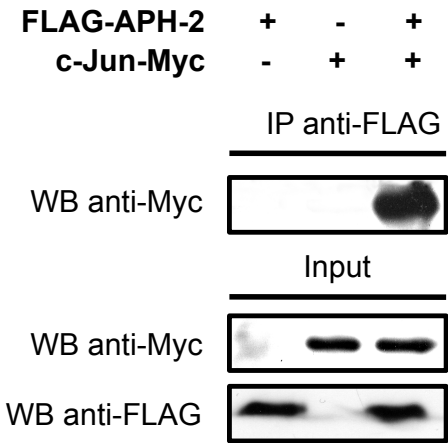

**B**

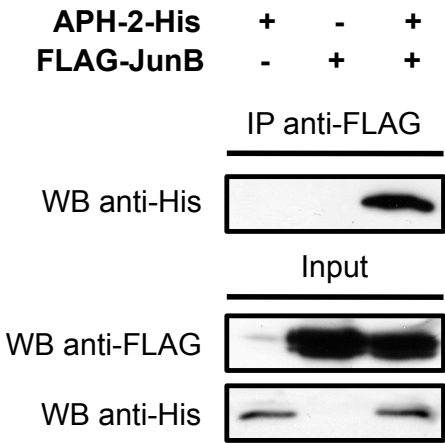

**C**

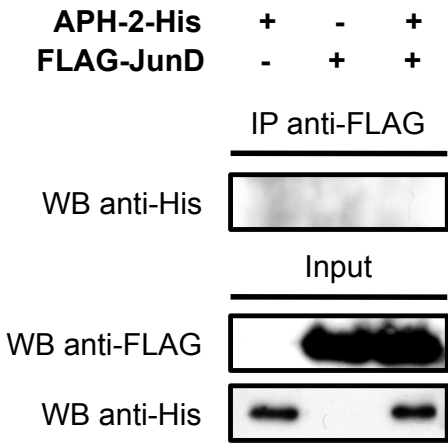

Supplement: Additional file 1 — APH-2 associates with c-Jun and JunB but not JunD. 293T cells were transiently transfected with the indicated expression plasmids. Two days after transfection, nuclear extracts were immunoprecipitated with the indicated antibodies (IP). The presence of proteins of interest in the immunoprecipitates was visualized by Western blot using the indicated antibodies (WB). (A) APH-2 interacts with c-Jun. (B) APH-2 binds JunB. (C) APH-2 does not interact with JunD. [file 1742-4690-9-98-S1.pdf]

**A**

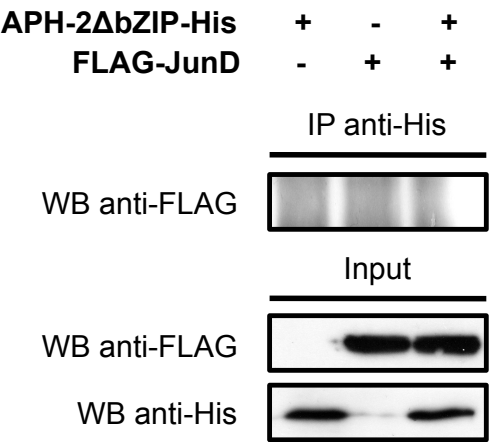

**B**

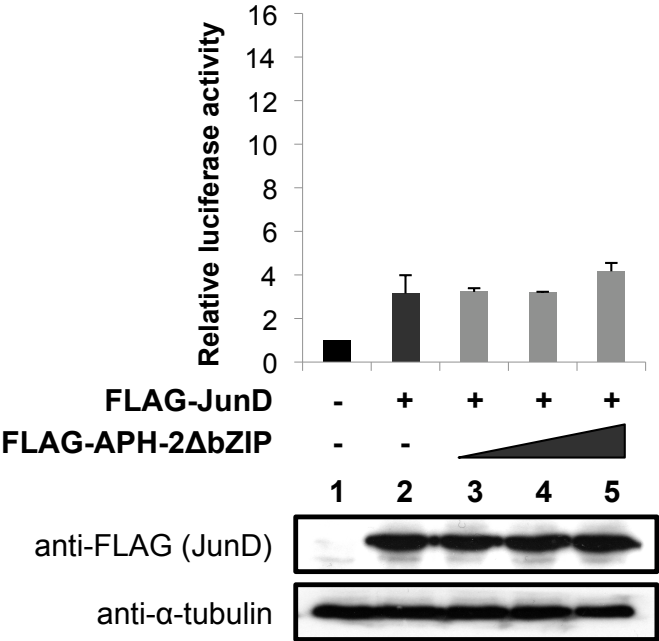

Supplement: Additional file 2 — APH-2ΔbZIP fails to stimulate JunD-mediated transcriptional activity. (A) APH-2ΔbZIP does not bind to JunD. Nuclear extracts from 293T cells transfected with the indicated vectors were prepared 48 hours post-transfection. Co-immunoprecipitations were then performed with His antibodies, followed by Western blotting with the indicated antibodies (WB). (B) APH-2ΔbZIP does not stimulate the transcriptional activity of JunD. The pAP-1 luciferase reporter construct was transiently co-transfected with the indicated expression vectors. Cells were lysed 48 hours post-transfection and processed for luciferase assays and Western blot analysis. The luciferase values represent an average of at least three independent experiments. [file 1742-4690-9-98-S2.pdf]

Additional File 3

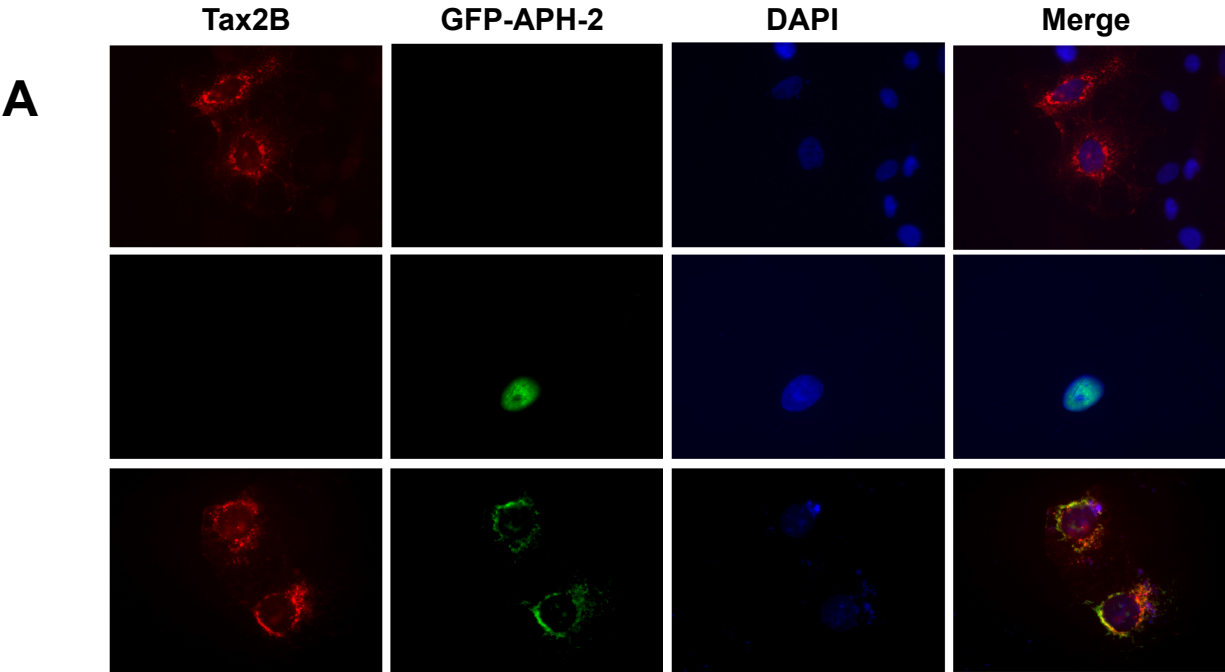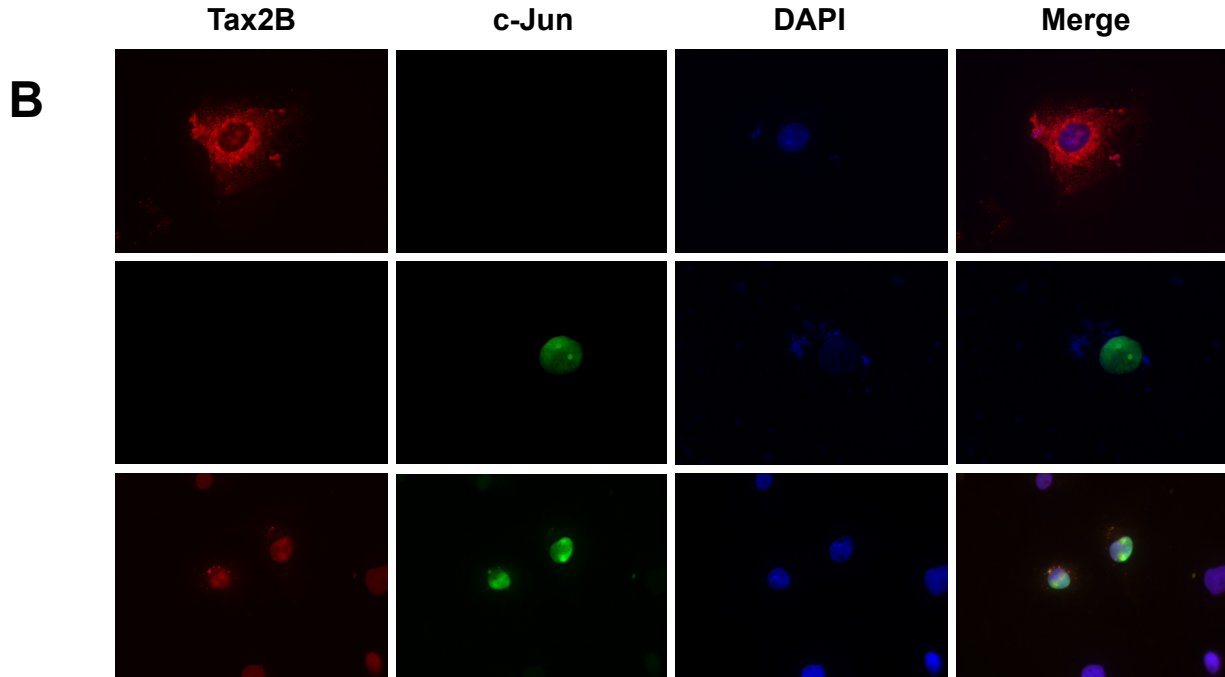

Supplement: Additional file 3 — Subcellular localization of APH-2 and c-Jun in the presence of Tax2B. COS-7 cells were transfected with the indicated plasmids. Cells were fixed and permealized 24 hours post-transfection. The proteins of interest were immunodetected and stained as indicated. Nuclei were stained with DAPI. Immunofluorescence images were obtained with a Zeiss Axio Imager microscope. Representative images of the entire cell population are shown. (A) Tax2B relocates APH-2 to the nuclear periphery. (B) c-Jun relocates Tax2B in the cell nuclei. [file 1742-4690-9-98-S3.pdf]

**A**

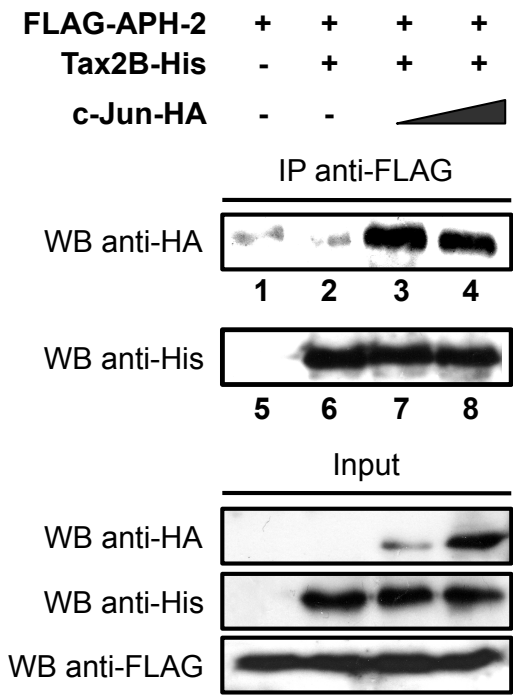

**B**

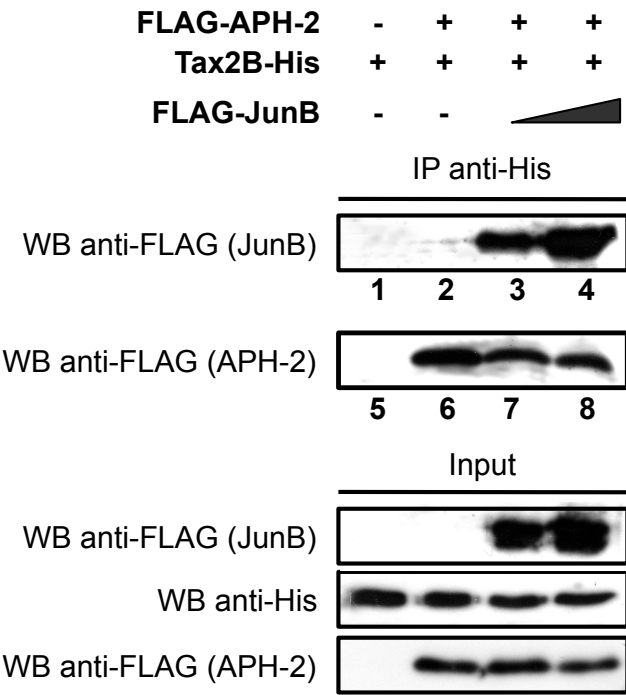

Supplement: Additional file 4 — c-Jun and JunB do not compete with Tax2B in its interaction with APH-2. Competition-binding assays were performed with nuclear extracts from 293 T cells overexpressing the indicated tagged-proteins. Co-immunoprecipitations were carried out using the indicated antibodies and the co-immunoprecipitated proteins were detected by Western blot using the indicated antibodies (WB). (A and B) c-Jun and JunB do not affect the interaction between APH-2 and Tax2B. [file 1742-4690-9-98-S4.pdf]

**A**

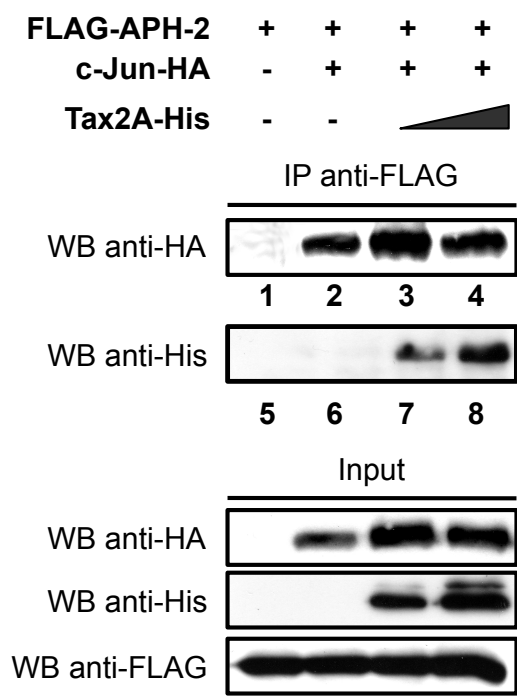

**B**

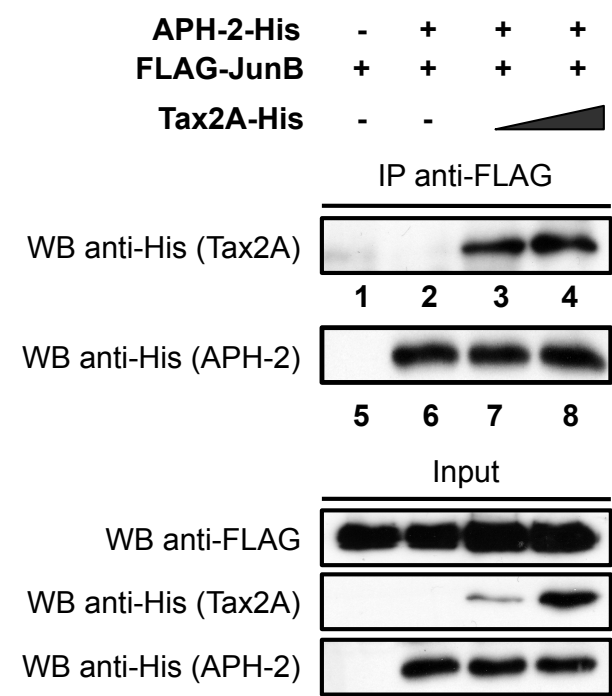

Supplement: Additional file 5 — APH-2 and c-Jun/JunB interaction is independent of Tax2A. (A and B) Tax2A does not affect the interaction between APH-2 and c-Jun/JunB. Competition-binding assays were performed with nuclear extracts from 293 T cells overexpressing the indicated tagged-proteins. Co-immunoprecipitations were carried out using FLAG antibodies and the co-immunoprecipitated proteins were detected by Western blot using the indicated antibodies. [file 1742-4690-9-98-S5.pdf]
